# Protease resistance of food proteins: a mixed picture for predicting allergenicity but a useful tool for assessing exposure

**DOI:** 10.1186/s13601-018-0216-9

**Published:** 2018-08-10

**Authors:** Jaap Akkerdaas, Muriel Totis, Brian Barnett, Erin Bell, Tom Davis, Thomas Edrington, Kevin Glenn, Gerson Graser, Rod Herman, Andre Knulst, Gregory Ladics, Scott McClain, Lars K. Poulsen, Rakesh Ranjan, Jean-Baptiste Rascle, Hector Serrano, Dave Speijer, Rong Wang, Lucilia Pereira Mouriès, Annabelle Capt, Ronald van Ree

**Affiliations:** 1 Department of Experimental Immunology, Academic Medical Center (AMC), Amsterdam, The Netherlands; 2 Bayer S.A.S, Bayer CropScience, Sophia Antipolis, France; 3 Formerly BASF Plant Science, Research Triangle Park, NC 27709 USA; 4 Regulatory Division, Product Characterization Center, Monsanto Co., St. Louis, MO USA; 5 DuPont Pioneer, DuPont Johnston Research and Development Center, Johnston, IA 50131 USA; 6 Syngenta Crop Protection, LLC, Research Triangle Park, NC 27709 USA; 7 Dow AgroSciences LLC, Indianapolis, IN 46268 USA; 8 Department of Dermatology and Allergology, University Medical Center Utrecht (UMCU), Utrecht, The Netherlands; 9 DuPont Haskell Laboratory, Newark, DE 19711 USA; 10 Allergy Clinic, Copenhagen University Hospital at Gentofte, Copenhagen, Denmark; 11 Bayer CropScience LP, Morrisville, NC 27560 USA; 12 Department of Medical Biochemistry, Academic Medical Center (AMC), Amsterdam, The Netherlands; 13 Health and Environmental Sciences Institute (HESI), Washington, DC 20005 USA; 14 Department of Otorhinolaryngology, Academic Medical Center (AMC), Amsterdam, The Netherlands

## Abstract

**Background:**

Susceptibility to pepsin digestion of candidate transgene products is regarded an important parameter in the weight-of-evidence approach for allergenicity risk assessment of genetically modified crops. It has been argued that protocols used for this assessment should better reflect physiological conditions encountered in representative food consumption scenarios.

**Aim:**

To evaluate whether inclusion of more physiological conditions, such as sub-optimal and lower pepsin concentrations, in combination with pancreatin digestion, improved the performance of digestibility protocols used in characterization of protein stability.

**Methods:**

Four pairs of established allergens and their related non/weakly-allergenic counterparts (seed albumins, muscle tropomyosins, plant lipid transfer proteins [LTP] and collagens) plus fish parvalbumin, were subjected to nine combinations of pH (1.2–2.5–4.0) and pepsin-to-protein ratio (PPR: 10–1–0.1 U/µg) for pepsin digestion, followed by pancreatin digestion in the presence of bile salts. Digestion was monitored by SDS-PAGE in conjunction with Coomassie staining and immunoblotting using rabbit antisera and human IgE.

**Results:**

At pH 4.0 and at PPR 0.1 most proteins, both allergen and non-allergen, were highly resistant to pepsin. Under conditions known to favor pepsin proteolysis, the established major allergens Ara h 2, Pru p 3 and Pen a 1 were highly resistant to proteolysis, while the allergen Cyp c 1 was not. However, this resistance to pepsin digestion only made Ara h 2 and to a lesser extent Pen a 1 and Pru p 3 stand out compared to their non-allergenic counterparts. Largely irrespective of preceding pepsin digestion conditions, pancreatin digestion was very effective for all tested proteins, allergens and non-allergens, except for Cyp c 1 and bovine collagen.

**Conclusions:**

Sub-optimal pH, low pepsin-to protein ratio, and sequential pepsin and pancreatin digestion protocols do not improve the predictive value in distinguish allergens from non-allergens. Digestion conditions facilitating such distinction differ per protein pair.

**Electronic supplementary material:**

The online version of this article (10.1186/s13601-018-0216-9) contains supplementary material, which is available to authorized users.

## Background

Allergenicity assessment is an important element of the overall risk assessment for genetically modified (GM) foods [1–4]. There is no single parameter that can predict whether a transgenic protein is going to behave as an allergen. When developing new GM traits, developers avoid the introduction of established allergens, and in the extension of that, also of molecules that may turn out to be cross-reactive with established allergens. Sequence databases such as AllergenOnline [5] or the COMPARE database [6] maintain a comprehensive list of molecules (and their primary sequences) that are reported to be allergens, based on proven IgE binding [7]. Sequence comparison of a candidate transgenic protein with sequences in an allergen database is an important step in the weight-of-evidence approach for allergenicity risk assessment [1–4, 8]. Another element to take into consideration in allergenicity risk assessment of a transgenic protein is its resistance to digestion by proteolytic enzymes from the gastro-intestinal (GI) tract [9–15]. The idea behind resistance to digestion as one of determinants of potential allergenicity is quite intuitive, since for readily digested proteins only small amount of the ingested protein molecule would reach the gut immune system in a sufficiently intact state and stimulate it to produce IgE antibodies or to trigger effector cells to induce allergic symptoms beyond the oral/esophageal tract. In this context, it is however important to realize that sensitization to food allergens may also occur via different routes than the gut such as the skin or the respiratory tract [16, 17], where resistance to pepsin or pancreatin is of no relevance. Moreover, if a rather labile protein is present at high concentrations, sufficient protein may survive proteolysis to cause systemic allergic symptoms. Thus, resistance to digestion is a parameter to consider in a weight-of-evidence approach to assess potential allergenicity risks.

The first report to systematically evaluate the utility of pepsin resistance testing as a parameter to help discriminate allergens from non-allergens was by Astwood et al. [9]. They evaluated 16 allergens and 9 non-allergens of plant origin using a protocol for pepsin digestion at pH 1.2 and a high pepsin-to-protein ratio (w/w: 19-fold). Unfortunately, this work did not clearly describe how many units of pepsin per weight of test protein this equates to. The conclusion from this study was that resistance to pepsin digestion clearly separates allergens from non-allergens. Several other reports followed up on those observations with conflicting results, demonstrating that not all food allergens are resistant to pepsin and not all non-allergens susceptible [15]. In part this is explained by the fact that the protocols used in these studies were not comparable, with different pH ranges (mainly ranging from 1.0 to 2.5, and only in a few cases pH 3.0 or 4.0), different pepsin to protein ratios (differing up to 4 orders of magnitude), and different incubation times. A number of studies also included pancreatin digestion in their assessment of a more limited number of molecules [15]. This brief review of conducted tests indicates that protocols differed significantly resulting in allergens being both resistant or susceptible proteins.

Apart from the fact that the lack of protocol standardization has made it difficult to reliably evaluate the discriminatory potential of resistance to pepsin digestion for allergenicity risk assessment, many of the studies have used conditions that have been criticized as not being representative of physiological conditions, i.e. too high pepsin-to-protein ratios, bias by optimal pH for pepsinolysis (around pH 2) while real life gastric pH values are often much higher (e.g. at young age or under influence of proton-pump inhibitors), not including pancreatin digestion, and not taking co-factors such as surfactants and food matrices into account [15].

To accommodate some of these criticisms, the objective of the present study was to compare resistance to GI digestion using a total of nine combinations of assay conditions of three different pHs during pepsin digestion (1.2, 2.5 and 4.0) and three pepsin-to-protein ratios (10, 1 and 0.1 U/µg), followed by pancreatin digestion with pancreatin in the presence of bile salts. Five established ‘complete allergens’ (shrimp tropomyosin [Pen a 1], peach lipid transfer protein [Pru p 3], peanut 2S albumin [Ara h 2], fish collagen and carp parvalbumin [Cyp c 1] and four of their respective functional non/low allergenic homologues (porcine tropomyosin, strawberry lipid transfer protein [Fra a 3], pea PA2 albumin and bovine collagen) were subjected to the nine digestion conditions and analyzed, using SDS-PAGE and Western blotting to evaluate stability, at 7 time-points. The overall aim was to accommodate some of the conditional variables that may constitute a more physiologically relevant platform for determining pepsin enzyme resistance.

## Materials and methods

### Antibodies

For detection of test proteins on immunoblot, rabbit polyclonal antisera instead of monoclonal antisera were used to increase the chance of recognition of peptides appearing during digestion. Five rabbit antibody reagents were commercially available: anti-porcine tropomyosin (Thermo Scientific, Waltham, MA, USA), anti-shrimp tropomyosin (Indoor Biotechnologies, Cardiff, UK), anti-fish collagen (Acris, San Diego, CA, USA), anti-bovine collagen (Biologo, Kronshagen, Germany), and anti-Ara h 2 (Indoor Biotechnologies, Cardiff, UK). Anti-pea albumin was custom produced with the same green pea albumin purified for this study (Charles River, Romans, France). Antiserum used for the detection of both peach and strawberry LTP was raised by immunization with recombinant apple LTP, resulting in broadly cross-reactive antibodies against plant LTPs, as described previously [18]. Rabbit antiserum against carp parvalbumin was raised by immunization with recombinant Cyp c 1 as described previously [19].

For IgE immunoblotting, serum samples with established IgE reactivity to Pru p 3/Fra a 3, Ara h 2, Pen a 1 and Cyp c 1, respectively, from an in-house reference serum bank were used. For both collagens, serum samples with IgE reactivity were not available. A panel of serum samples (n = 12) from green pea sensitized subjects was screened for IgE reactivity against pea PA2 albumin, but none of the samples recognized the protein.

### Purified proteins

Five out of nine purified proteins were purchased commercially: pig tropomyosin (Sigma, St. Louis, MO, USA), bovine collagen type 1 (ThermoFisher Scientific, Rockford, IL, USA), fish collagen type 1 (Eonova-coll, Debrecen, Hungary), shrimp tropomyosin and peanut Ara h 2 (Indoor Biotechnologies). Pea PA2 albumin was purified from dried green peas as described by Vioque et al. [20]. Carp parvalbumin was purified from fresh carp muscle as described by Kuehn et al. [21]. Both strawberry and peach LTP were expressed in *Escherichia coli* Rosetta-gami 2 (DE3) pLysS strain as described previously [18, 22]. In short, bacteria were grown at 37 °C, 200 rpm until the OD_600_ reached 0.6. Expression of LTP was induced by adding 1 mM IPTG, followed by 5 h of culturing at 30 °C. Subsequently cells were lysed by addition of lysozyme and then sonicated; following centrifugation, the supernatant was used for purification of both recombinant LTPs. Purification was based on ion exchange chromatography (IEC) using an ÄKTA ™ Purifier 10 system (GE Healthcare, Hoevelaken, NL): first a separation on an SP Sepharose FF column (cation exchanger), then a desalting step on a HiPrep 26/10 desalting column, followed by a second separation on a MonoQ 5/50 GL column (anion exchanger). For Fra a 3, separation on an extra MonoS column (cation exchanger) was added as a last IEC step. The final purified preparations were concentrated by ultrafiltration over a 3 kDa (YM-3) cut-off filter (Amicon, Darmstadt, Germany).

All purified proteins (see Table 1) were aliquoted in 400 µl portions and stored at − 20 °C until further use, at a protein concentration of 1 mg/ml (water), except the collagens that were stored at 2 mg/ml. Percent identity of pairs of proteins were determined using Clustal W.

**Table 1:** Protein pairs and their degree of identity

Protein family	Allergens	MW on SDS-PAGE	Non-/weak allergens	MW on SDS-PAGE	% identity
Lipid transfer proteins	Peach Pru p 3	9 kDa	Strawberry Fra a 3	9 kDa	66.7
Albumins	Peanut Ara h 2	17 kDa	Pea PA2 albumin	25 kDa	5.2
Tropomyosins	Shrimp Pen a 1	36 kDa	Porcine tropomyosin	33 kDa	55.0
Collagens	Fish collagen type 1	4 bands (> 90 kDa)	Bovine collagen type 1	4 bands (> 90 kDa)	55–75
Parvalbumins	Carp Cyp c 1	12 kDa			

### Combined pepsin and pancreatin digestion protocol

For pepsin digestion, three concentrations of pepsin 10, 1, and 0.1 U/µg test protein were made in solutions of 0.2% NaCl that were adjusted to either pH 1.2, 2.5, or 4.0, resulting in a total of 9 assay conditions. The pepsin stock solution (Sigma) had a specific activity of 3850 U/mg as provided by the manufacturer. For both collagens the pepsin concentrations were doubled to have the same pepsin-to-protein ratios. For each protein, a total of nine different simulated gastric fluids (SGF) were used. SGF without test protein (1710 µl) was pre-incubated in a water bath for 5 min at 37 °C, after which 90 µl of aliquoted test protein was added. For each time point a separate tube was used for digestion: t = 0 (sample G0), t = 5 min (G5), t = 10 min (G10) and t = 60 min (G60). At these time points, pH was adjusted to pH 7.5 using 1 M NaOH, to inactivate the pepsin. For the G0 sample, inactivation was done before addition of the test protein. Before adding pancreatin for the pancreatin digestion phase, bile salt solution in 50 mM KH_2_PO_4_/K_2_HPO_4_ was added resulting in a final concentration of 3.2 mM KH_2_PO_4_/K_2_HPO_4_ and 4 mM of both sodiumtaurocholate and sodiumglycodeoxycholate (both from Sigma).

**Fig. 1:**
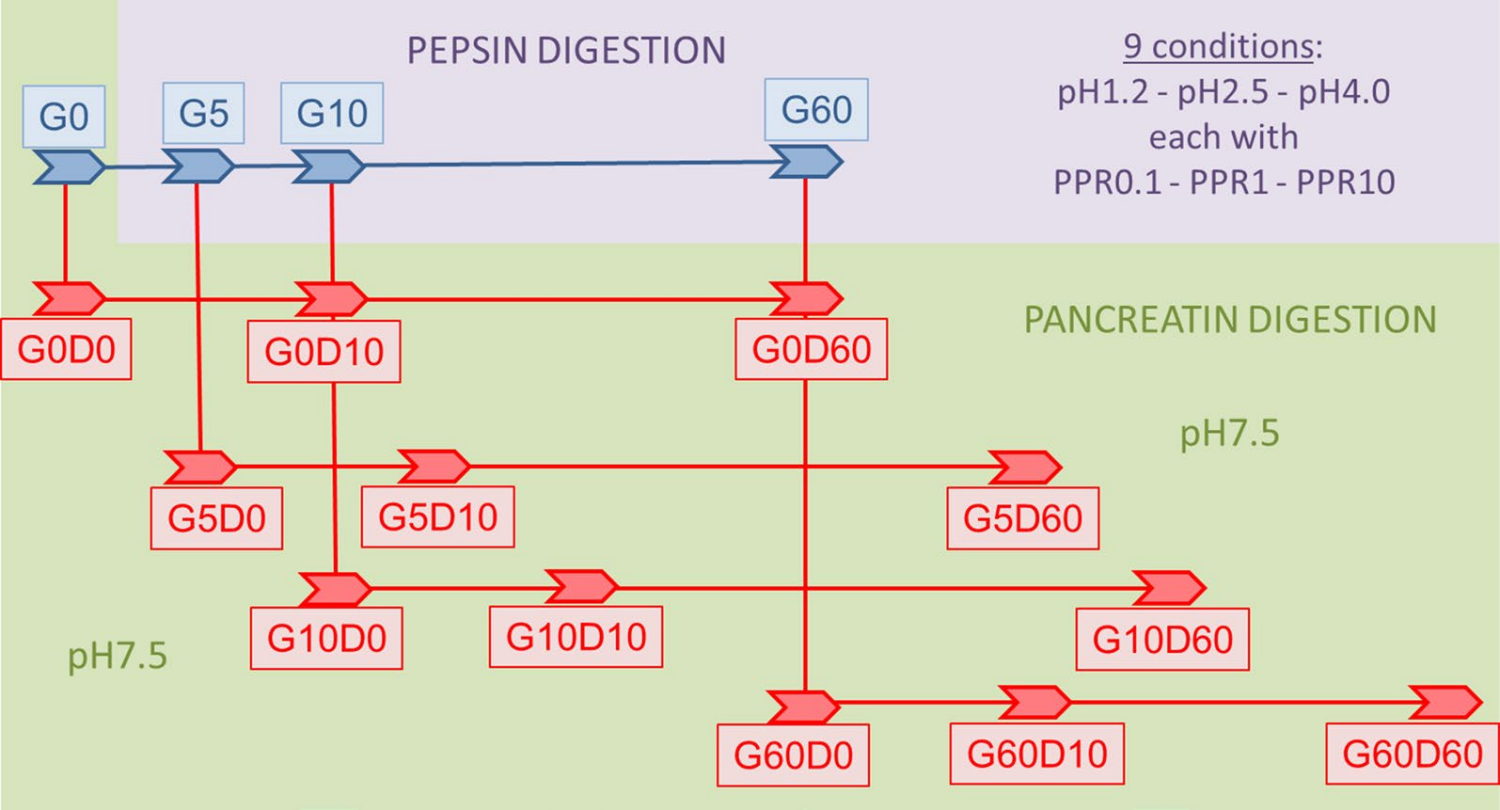
A schematic representation of the sequential sampling during the combined gastric and duodenal digestion protocol. G stands for gastric, D for duodenal. The numbers indicate the time at which samples were taken in minutes. G0 and G0D0 samples have not been exposed to acidic pH. In total 9 different combinations of pH and pepsin-to-protein ratios were tested in the gastric phase. In combination with the duodenal phase per molecule a total of 144 samples (4 gastric plus 3 × 4 duodenal times 9 conditions) were analyzed by SDS-PAGE and immunoblotting

Each of the remaining pepsin digestion samples (with inactivated pepsin and bile salts) then served as starting point for the pancreatin digestion. First, a 100 µl sample was drawn from the four tubes representing the different time points of the pepsin phase, and to this sample inactivated (10 min at 90 °C) pancreatin (Sigma) was added (6.5 µl of 1% w/v in 50 mM KH_2_PO_4_/K_2_HPO_4_ at pH7.5), giving the 4 different t = 0 samples for pancreatin digestion after pepsin digestion: G0D0, G5D0, G10D0 and G60D0. To the remaining samples 115 µl of the same, but active pancreatin solution was added. Pancreatin digestion was monitored at two time-points, i.e. 10 and 60 min. At both time points, 100 µl samples were drawn that were immediately inactivated by adding a mix of NuPAGE sample buffer and NuPAGE reducing agent (see below). This resulted in the following samples: G0D10 and G0D60, G5D10 and G5D60, G10D10 and G10D60, and G60D10 and G60D60. An overview of the sampling scheme is presented in Fig. 1.

For SDS-PAGE and Western blotting 100 µl samples were drawn, which were mixed with NuPAGE LDS sample buffer and NuPAGE reducing (Invitrogen, Carlsbad, CA, USA) in a ratio (v/v/v) of 13:5:2. All samples for SDS-PAGE and Western blotting analyses were heated for 10 min at 70 °C and then stored at − 20 °C until use.

### SDS-PAGE and Western blotting

Proteins (18 μl sample per lane) were separated by SDS-PAGE (NuPAGE^®^ 4–12% Bis-Tris gel; NuPAGE^®^ 3–8% for the collagens; Invitrogen) and Western blotting was performed by transferring the proteins semi-dry to nitrocellulose on a Novablot electrophoretic transfer apparatus, according to the protocol of the manufacturer (Invitrogen). After blocking with PBS/5% skim milk powder/0.1% Tween-20 for a minimum of 10 min, the blots were incubated overnight with appropriate dilutions of polyclonal rabbit antisera in 12.5 ml of PBS/0.1% Tween-20/0.5% skim milk powder. After washing 5 times (PBS/0.1% Tween-20), blots were incubated (4 h) with IRDye800CW-labeled goat anti-rabbit IgG (Licor Biotechnology, NE, USA) and subsequently washed as before. The IRDye800-labeled proteins were detected by infrared scanning using Odyssey V3.0 scanning software (Westburg, Leusden, The Netherlands).

## Results

### Lipid transfer proteins

**Fig. 2:**
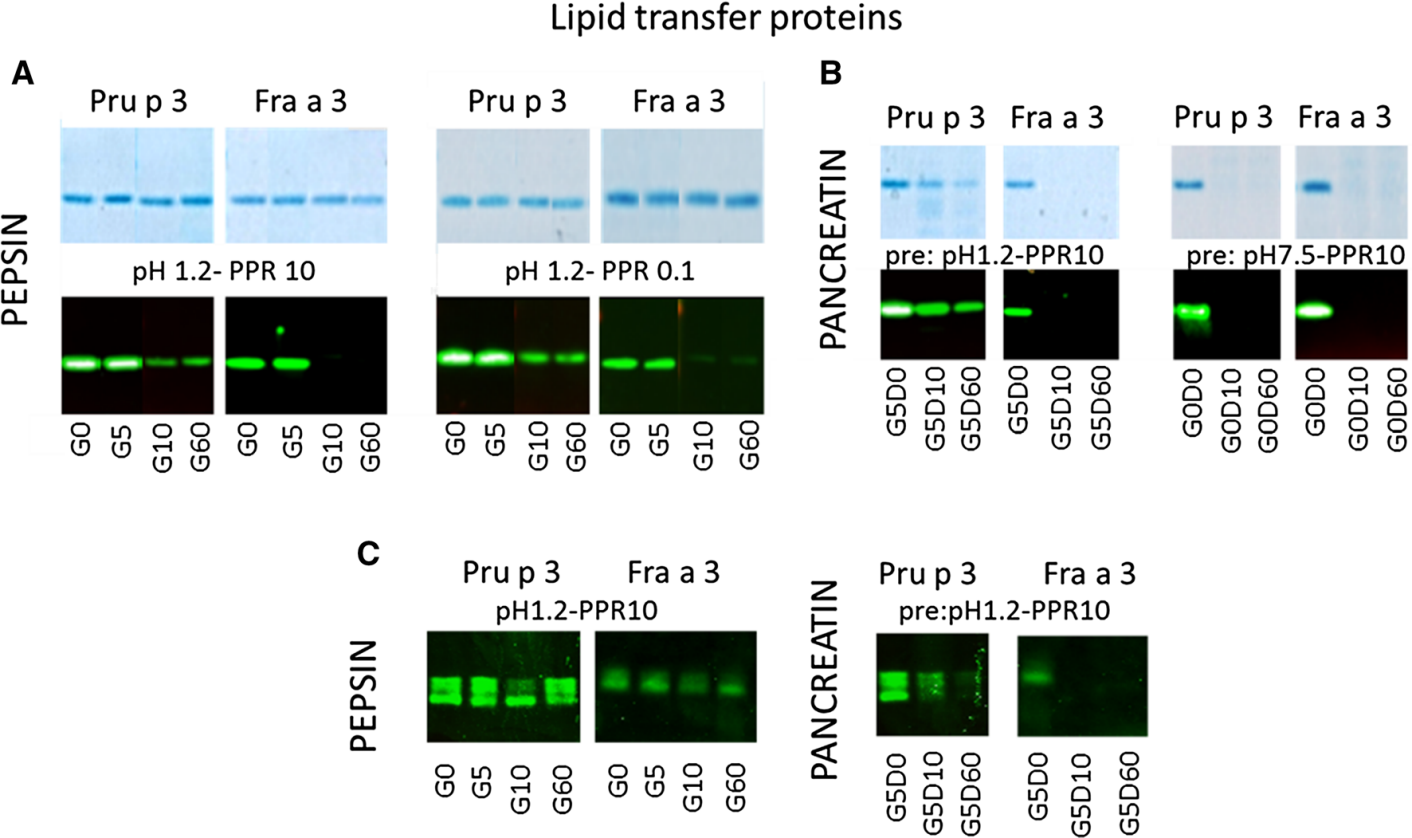
Selected SDS-PAGE and immunoblot samples are shown for both LTPs, Pru p 3 and Fra a 3. **A** Both proteins are highly resistant to pepsin as judged by SDS-PAGE. In contrast, on immunoblot the intensity of recognition by rabbit IgG clearly decreases at t = 10 and t = 60 min. There is a suggestion that this decrease is more significant for Fra a 3, but it cannot be excluded that this is more a result of properties of the rabbit antiserum than of the proteins. In support of this the G0 band of Pru p 3 is more intense than of Fra a 3. **B** Both proteins not having been exposed to acidic pH are readily digested by pancreatin. When pre-exposed to low pH, Pru p 3 displays significantly higher resistance to pancreatin than its homologue Fra a 3. **C** Also when IgE is used for immunoblotting both LTPs display high resistance to pepsin. The higher resistance of Pru p 3 to pancreatin is also observed with IgE

Lipid transfer proteins (LTP) from peach and strawberry were obtained as purified recombinant proteins, Pru p 3 and Fra a 3, respectively, with the former being an established major allergen and the latter a weak allergen [22, 23]. Overall, both LTPs were equally resistant to pepsin digestion at all nine combinations of pH and pepsin-to-protein ratios (PPRs) when judged by SDS-PAGE in conjunction with Coomassie staining (Fig. 2A). However, at time points 10 and 60 min (pH 1.2 and 2.5) rabbit IgG antibody staining showed a decrease in detection for both peach and strawberry LTP, again at all nine pHs and PPRs. This loss of antibody binding appeared to be greater for Fra a 3 (shown for pH1.2 with PPR 10 and 0.1 in Fig. 2A), although it cannot be fully excluded that this is (in part) explained by lower sensitivity of the rabbit antiserum for Fra a 3 compared to Pru p 3.

Exposure to pepsin for 5 min at pH 1.2 irrespective of the PPR made Fra a 3 highly susceptible to subsequent pancreatin digestion, as judged by both SDS-PAGE and immunoblotting. Although some digestion of Pru p 3 by pancreatin also occurred, this was only observed following exposure to the highest pepsin concentration (Fig. 2B). Both LTPs were completely digested by pancreatin after 10 min, following pepsin digestion at pH 2.5 and at pH 4.0, with any PPR. This was even true for the associated negative control samples for pepsin digestion (G0D0) that were neutralized (pH 7.5) prior to addition of LTP, i.e. they had not been exposed to acidic pH conditions at all. At the first time point of pancreatin digestion (G0D10), both Fra a 3 and Pru p 3 in these negative control samples were undetectable on SDS-PAGE and by immunoblotting (shown for PPR 10 in Fig. 2B).

Some digesta were also used for IgE immunoblotting (Fig. 2C). The complete disappearance of antibody binding to Fra a 3 after 10 min exposure to pepsin (PPR 10) was not observed using IgE. On the other hand, the higher susceptibility of Fra a 3 to subsequent pancreatin digestion was confirmed using IgE antibodies.

### Albumins

**Fig. 3:**
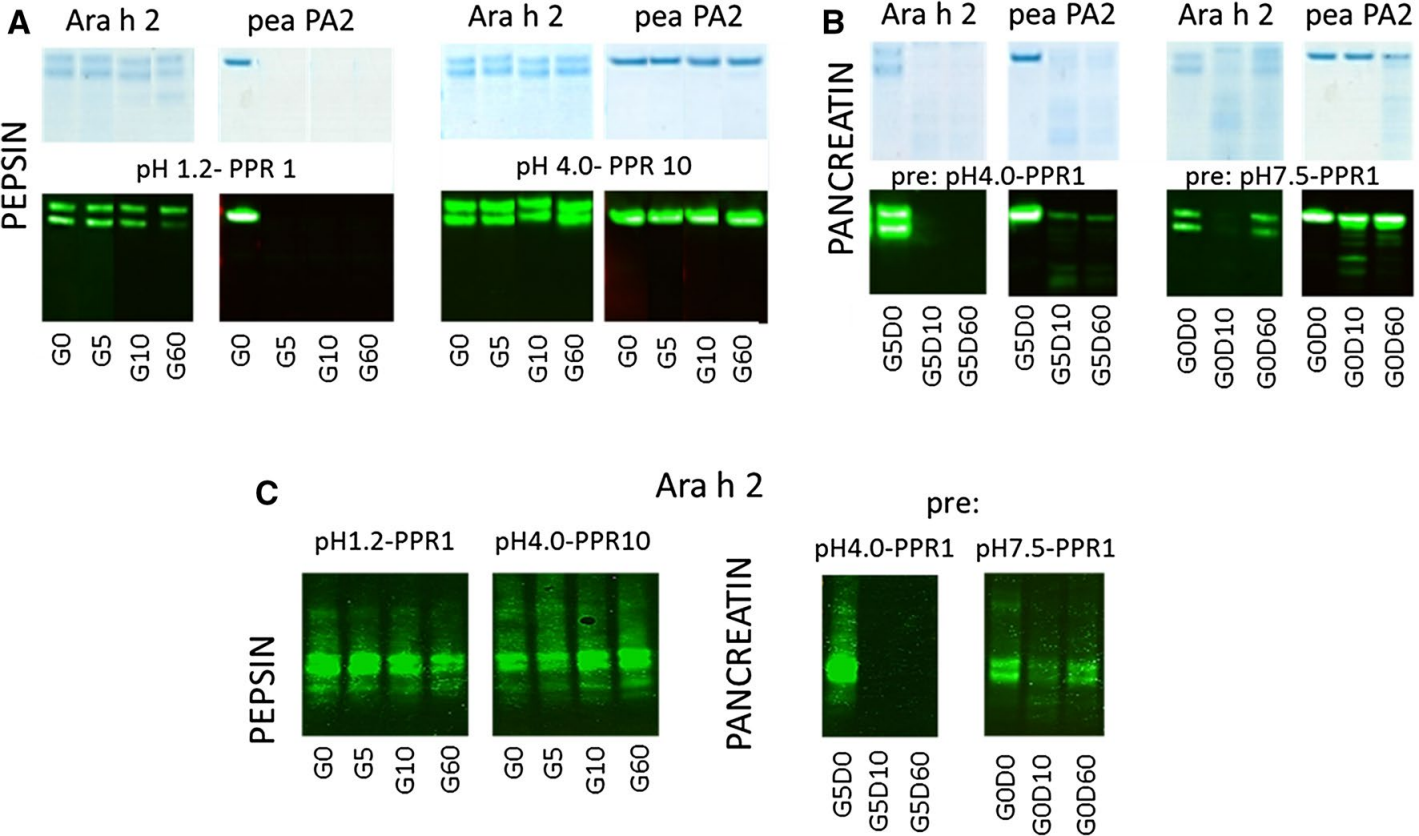
Selected SDS-PAGE and immunoblot samples are shown for both albumins, Ara h 2 and pea PA2 albumin. **A** Ara h 2 displays complete resistance up to 1 h to pepsin at acidic pH, whereas the pea albumin is undetectable after 5 min. Both albumins are unaffected by pepsin at pH 4.0. **B** Pea albumin PA2 appears to be more resistant to pancreatin than Ara h 2. Surprisingly the disappearance of Ara h 2 is transient when it has not been exposed to acidic pH and active pepsin. **C** High resistance of Ara h 2 to pepsin is confirmed for IgE. For pea albumin susceptibility could not be confirmed because no sera with pea albumin reactive IgE were found. Susceptibility characteristics of Ara h 2 to pancreatin were also confirmed with IgE

Purified natural peanut 2S albumin Ara h 2, an established allergen, and green pea PA2 albumin (PA2), a weak/non-allergen are both abundant seed storage proteins, but have no significant sequence homology (5%). At pH 1.2 with PPR 10 and 1, PA2 was not detected after 5 min of pepsin digestion, both on SDS-PAGE and immunoblot (shown for PPR 1 in Fig. 3A). At PPR 0.1 truncated bands of PA2 remained detectable up to 1 h of pepsin digestion (Additional file 1: Fig. E1a). Ara h 2 was highly resistant to pepsin digestion at pH 1.2, with the higher band of the Ara h 2 doublet staying visible up to 1 h, even with the highest dose of pepsin (Additional file 1: Fig. E1b). At PPR1, the Ara h 2 doublet remained intact (Fig. 3A). Ara h 2 appeared slightly more susceptible to pepsinolysis at pH 2.5 than at pH 1.2 (Additional file 1: Fig. E1c). In contrast, pea albumin was clearly more resistant at pH 2.5 than at pH 1.2 with the PPRs 10 and 1 (shown for PPR 1 in Additional file 1: Fig. E1d). At pH 4.0, for both albumins no significant digestion was observed (Fig. 3A).

Pepsin digestion of Ara h 2 at pH 2.5 and pH 4.0 made it highly susceptible to pancreatin digestion. At all three PPRs the protein doublet had completely disappeared at 10 min in both SDS-PAGE and immunoblotting (shown for pH 4.0/PPR 1 in Fig. 3B). Pepsin digestion at pH 1.2 (but also in the negative control samples that had not been exposed to acidic pH) followed by pancreatin digestion resulted in an unexpected observation: after 10 min in pancreatin, the Ara h 2 doublet almost disappeared, but at 60 min the bands increased in intensity again. This phenomenon was observed reproducibly independent of the preceding PPR, but was most obvious at PPR 1 (Fig. 3B). Judged by immunoblotting, PA2 was slightly more resistant to pancreatin digestion than Ara h 2 (shown for PPR 1 in Fig. 3B).

IgE immunoblotting confirmed the high resistance of Ara h 2 to pepsin digestion (shown for pH 1.2/PPR 1 in Fig. 3C). Also, for IgE antibodies, pancreatin exposure of Ara h 2 following pepsin digestion at pH 4 resulted in complete disappearance of IgE binding. Finally, the reduction and subsequent increase of antibody binding observed for negative control samples (G0D0) under influence of pancreatin was also observed for IgE antibodies, albeit less convincingly (Fig. 3C).

### Tropomyosins

**Fig. 4:**
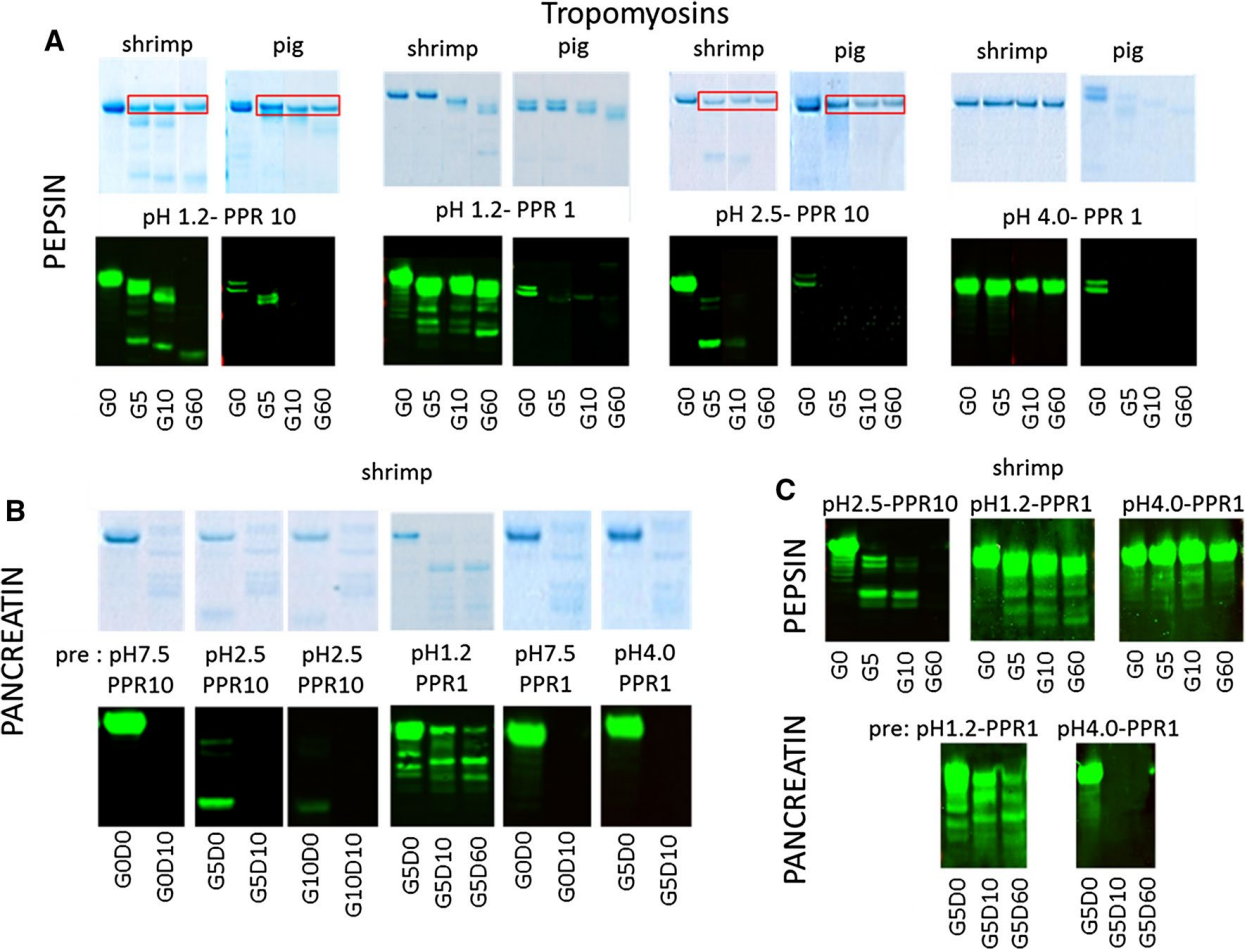
Selected SDS-PAGE and immunoblot samples are shown for both tropomyosins, from shrimp (Pen a 1) and pig. **A** At pH 1.2 and 2.5 judged by SDS-PAGE no very clear difference in resistance is apparent, although for shrimp tropomyosin the presence of breakdown peptides at PPR 10 appears to be more prominent. Pepsin bands visible at PPR 10 are boxed in red. At PPR 1 resistance of both tropomyosins appears quite high. Immunoblot analyses of gastric samples exposed to pH 1.2 and 2.5 confirm higher stability of shrimp tropomyosin but it cannot be excluded that this is rather a result of different affinities of rabbit antisera. The most convincing difference between allergen and non-allergen is observed at pH 4.0, with shrimp tropomyosin being totally unaffected and pig being readily digested. **B** Shrimp tropomyosin is highly susceptible to pancreatin except when having been pre-exposed to pH 1.2. **C** IgE immunoblotting confirms pepsin and pancreatin resistance characteristics observed wit rabbit IgG

Shrimp tropomyosin Pen a 1, an established allergen, was truncated by pepsin at pH 1.2 with PPR 10 and PPR 1 at 5 and 10 min, followed by further truncation at 60 min (Fig. 4A). Truncated peptides slightly below the molecular mass of the native protein as well as smaller peptides cleaved off were still detected on immunoblot. At pH 1.2 /PPR 0.1 no cleavage was observed (Additional file 2: Fig. E2a). At pH 2.5, Pen a 1 was much more susceptible to digestion (shown for PPR 10 in Fig. 4A), with truncation observed even at PPR 0.1 (Additional file 2: Fig. E2b). At pH 4.0, with all three PPRs, Pen a 1 was resistant to pepsin digestion (shown for PPR 1 in Fig. 4A).

Pig tropomyosin, a weak/non-allergen, was also truncated by pepsin at pH 1.2/PPR 10, and was not detected by antibodies at 10 min into the pepsin digestion (Fig. 4A). At PPR 1, weak recognition of a truncated band remained present up to 60 min (Fig. 4A). At PPR 0.1, some truncation was only observed after 60 min (Additional file 2: Fig. E2c). At pH 2.5, pig tropomyosin was fully digested at PPR 10 (Fig. 4A), progressively truncated at PPR 1 with only very weak residual antibody recognition at 5 min, and almost unaffected at PPR 0.1 with only slight truncation at 60 min (not shown). At pH 4.0 and PPR 10 or PPR 1, the protein was rapidly digested with no antibody recognition (shown for PPR 1 in Fig. 4A). At pH 4.0/PPR 0.1 some minor truncation was observed, but significant antibody binding persisted (not shown).

Pig tropomyosin was fully digested by pancreatin irrespective of the preceding pepsin digestion conditions (not shown). This was also true for pancreatin digestion of shrimp tropomyosin (shown for pH 2.5/PPR 10 and pH 4.0/PPR 1 in Fig. 4B), except when following pepsin digestion at pH 1.2 in combination with the two higher PPRs. Under those conditions, truncation was observed, with recognition of some lower molecular mass bands on immunoblot up to 60 min of pancreatin digestion (Additional file 2: Fig. E2d).

IgE immunoblotting of shrimp tropomyosin pepsin digestion samples gave comparable results as observed with rabbit antisera (Fig. 4C).

### Collagens

**Fig. 5:**
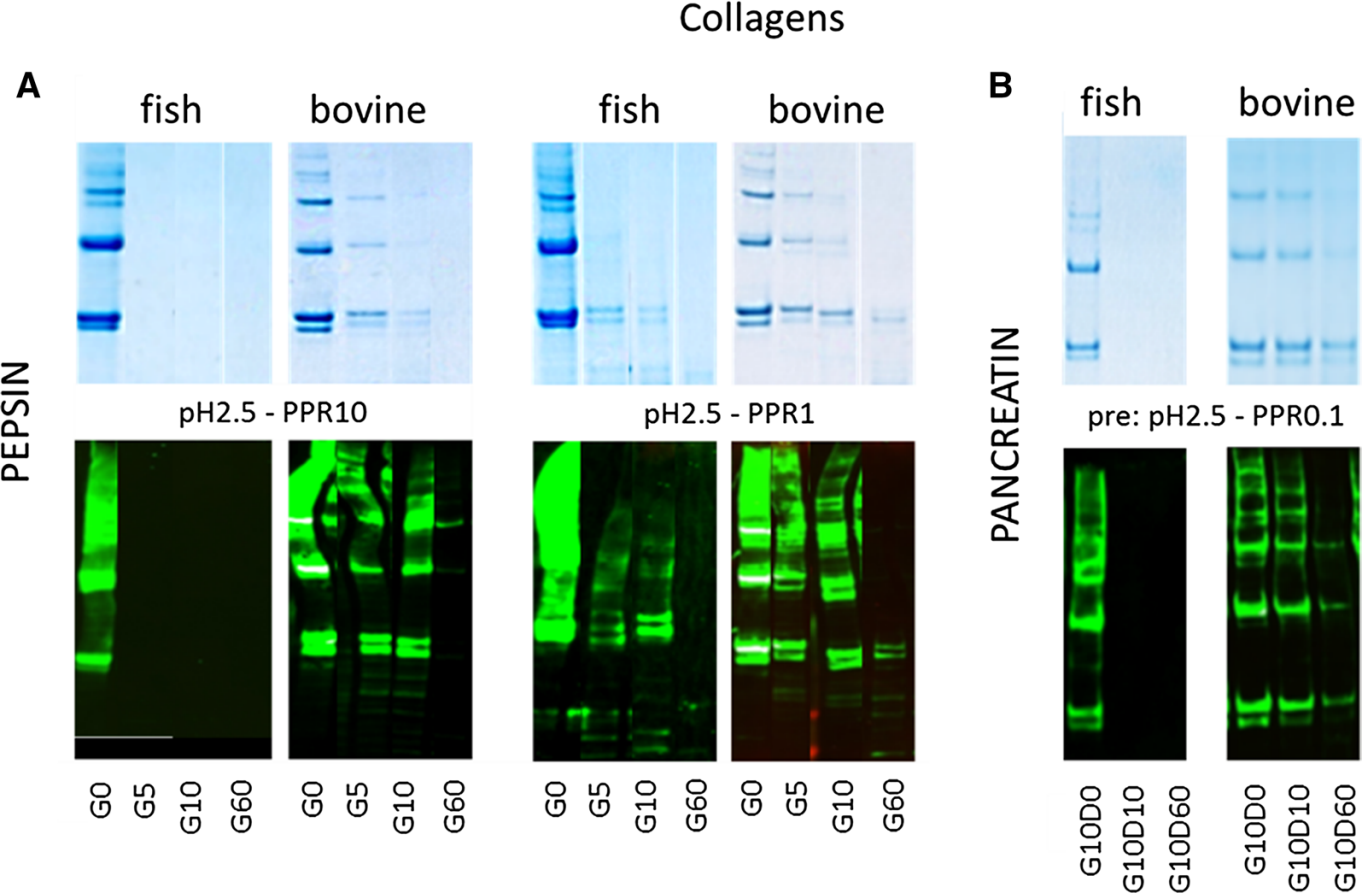
Selected SDS-PAGE and immunoblot samples are shown for both collagens from fish and beef. **A** Fish collagen is more resistant to pepsin than bovine collagen. **B** Fish collagen is highly susceptible to pancreatin whereas bovine is quite resistant

Fish collagen, mainly reported as an allergen in Japanese fish allergic patients [24, 25], was effectively digested by pepsin at the highest PPR, independent of pH (shown for pH 2.5 in Fig. 5A). At lower PPRs resistance increased, both on SDS-PAGE and immunoblot (shown for pH2.5/PPR 1 in Fig. 5A). Bovine collagen, rarely reported to be allergenic [26], was significantly more resistant to pepsin digestion, with no visible digestion at PPR 0.1 (Additional file 3: Fig. E3). Only at pH 1.2 and in particular pH 2.5 some digestion was observed with PPR 10 (Fig. 5A).

Fish collagen was very susceptible to pancreatin digestion, irrespective of the preceding gastric conditions (shown for pH 2.5/PPR0.1 in Fig. 5B). Bovine collagen on the other hand was quite resistant to pancreatin digestion, in particular when preceded by pepsin digestion at pH 2.5-PPR 0.1 (Fig. 5B).

### Parvalbumin

**Fig. 6:**
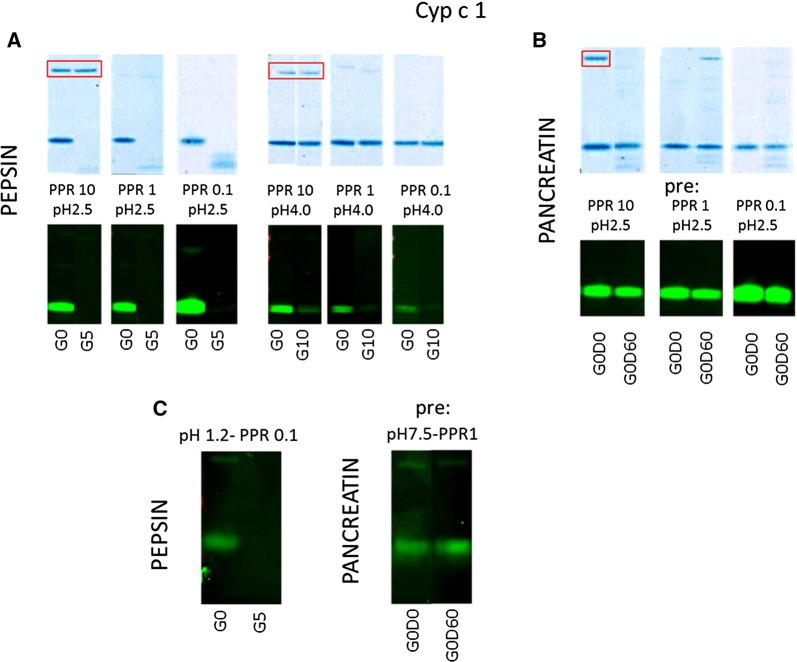
Selected SDS-PAGE and immunoblot samples are shown for carp parvalbumin, Cyp c 1. **A** At pH 2.5 Cyp c 1 is readily digested by pepsin. With decreasing PPR, presence of residual breakdown peptide increases, but these are not recognized on immunoblot. Pepsin band on SDS-PAGE is boxed in red. **B** Cyp c 1 is highly resistant to pancreatin with some appearance of similar breakdown peptides as observed upon exposure to pepsin. **C** IgE immunoblotting confirms pepsin and pancreatin resistance characteristics observed wit rabbit IgG

Carp parvalbumin, an established allergen, was very susceptible to pepsin digestion at pH 1.2 and 2.5, with complete disappearance of the native protein band on SDS-PAGE and with complete loss of antibody binding on immunoblot at all three PPRs (shown for pH 2.5 in Fig. 6A). On SDS-PAGE, with the lower PPRs some cleavage peptides were visible at three molecular masses below the native protein, but these were not recognized by antibodies. At pH 4.0 no decrease in band intensity occurred on SDS-PAGE, but that of antibody binding decreased at 10 and 60 min (Fig. 6A). In contrast to the susceptibility to pepsin, carp parvalbumin proved to be highly resistant to pancreatin digestion both on SDS-PAGE and immunoblot with rabbit IgG (shown for pH 2.5 in Fig. 6B) and with human IgE (Fig. 6C). Susceptibility to pepsin digestion of IgE binding to parvalbumin was confirmed (shown for pH1.2 /PPR 0.1 in Fig. 6C).

## Discussion

Stability to digestion is one of the components of the weight-of-evidence approach for allergenicity risk assessment of novel food proteins. The present study investigated whether distinction of food allergens and non/weakly allergenic food proteins can be achieved with currently applied digestion protocols using optimal conditions for pepsinolysis, and whether performance would improve by using less optimal physiologically occurring conditions, and by sequentially extending such protocols with pancreatin digestion. A schematic overview of the outcome of these studies is given in Table 2, essentially showing that there is no single protocol that allows distinction between allergens and non-allergens based on resistance to digestion.

**Table 2:**
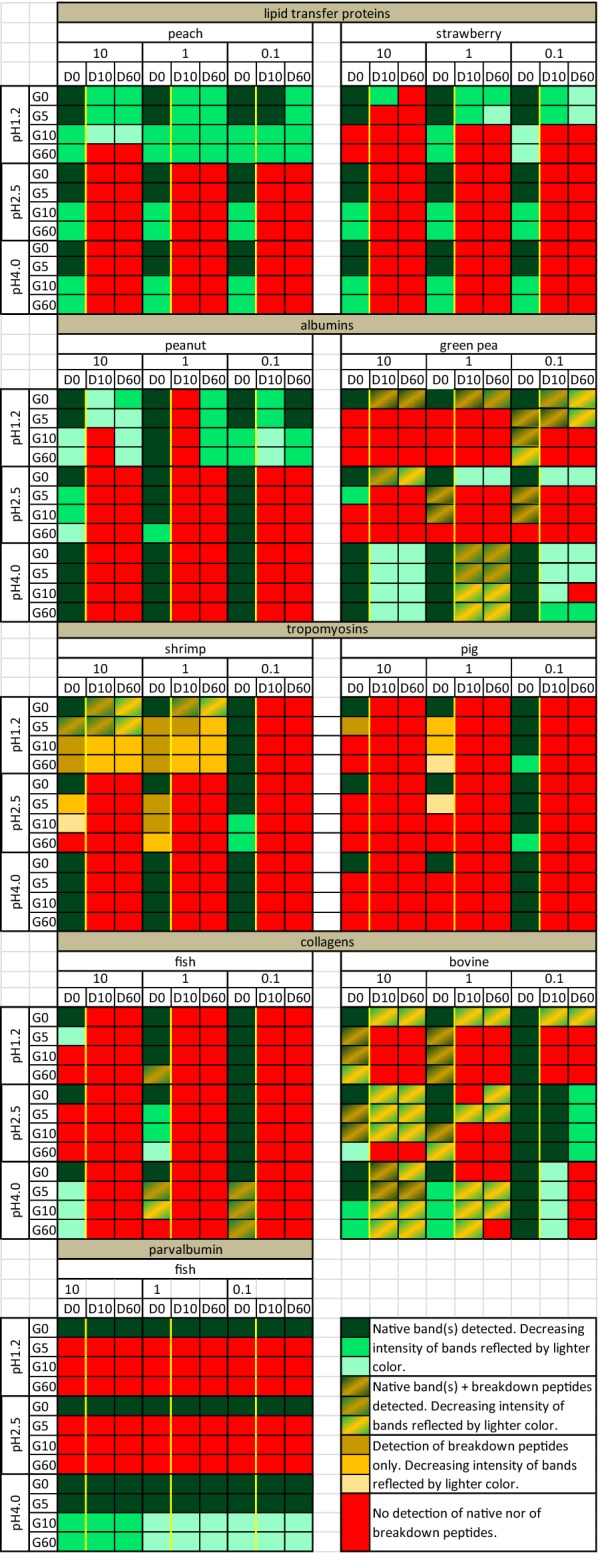
Schematic overview of resistance to digestion judged by immunoblot

For the pepsin phase under optimal conditions (pH 1.2/2.5–PPR 10/1), a quite mixed picture emerges (Table 2) with two established major allergens, Pru p 3 and Ara h 2, being highly resistant to pepsin digestion (Figs. 2A, 3A). Alternatively, full-length shrimp tropomyosin was quite susceptible to pepsin digestion, but resulting breakdown peptides retained antibody-binding capacity, both rabbit IgG (Fig. 4A) and human IgE (Fig. 4C). The fourth major allergen, fish parvalbumin, was highly susceptible to pepsin digestion (Fig. 6A).

In the current study, optimal (low) pH with saturating pepsin concentrations clearly distinguished allergen from non-allergen in case of Ara h 2 and pea PA2 albumin, and to a lesser extent both LTPs and tropomyosins (Table 2). In fact, for tropomyosins distinction was best made at pH 4.0–PPR 1 (Fig. 4A). For the collagens, the one with most convincing evidence for allergenicity, fish collagen, turned out to be much more susceptible to pepsin than bovine collagen (Fig. 5A). For fish parvalbumin we did not have a comparison to a poorly allergenic homologue, but the well-established strong allergen carp parvalbumin was highly sensitive to pepsin digestion (Fig. 6A, C), i.e. not fitting the hypothesis that major allergens are pepsin resistant.

A very consistent observation was that pepsin digestion at the higher pH of 4.0 is very ineffective, both for the established allergens and their less allergenic counterparts. Exceptions to these observations were pig tropomyosin and fish collagen, showing breakdown and loss of antibody binding at pH 4.0 with PPR 10 and 1. A pepsin-to-protein ratio of 0.1 was ineffective for digestion of all tested proteins. Overall, therefore, pepsin digestion assays conducted at pH 4.0 and PPR 0.1 are, despite in some ways perhaps being more physiological, not of added value to distinguish allergens from non-allergens: Most proteins are poorly processed under these conditions, irrespective of being an allergen or not. On the other hand, this means that at higher pH, such as occurring during the use of proton-pump inhibitors, more intact protein will reach the intestines. This may translate into a higher risk of allergic symptoms, but this will of course also depend on the subsequent behavior during pancreatin digestion.

Pancreatin digestion is currently not routinely combined with pepsin digestion in protocols applied for allergenicity risk assessment. Here we have tested this combination for all nine conditions of pepsin digestion. Carp parvalbumin that only survived pepsin digestion at pH 4.0 was completely resistant to pancreatin digestion, a finding that seems to be in accordance with in vivo studies [27]. Quite surprisingly, the other three established allergens Ara h 2, Pru p 3 and Pen a 1 were highly susceptible to pancreatin after having survived pepsin digestion at pH 2.5 and pH 4.0 in combination with all three PPRs (Table 2). All three allergens were completely digested at the first time-point (10 min) of the pancreatin phase. In terms of using the addition of the pancreatin digestion as a means of distinguishing allergens from their non-allergenic counterparts this did however not really help. Examples of this inconsistency include Fra a 3 and pig tropomyosin which were equally susceptible as their allergenic counterparts, and the expected low allergenic pea albumin appeared slightly more stable than the peanut protein, Ara h 2.

After pre-treatment with pepsin at pH 1.2 variable resistance to pancreatin digestion was observed (Table 2). Pru p 3 showed a moderate decrease only after pre-treatment with the highest pepsin concentration, but was unaffected by pancreatin after PPRs 1 and 0.1. This clearly distinguishes this major allergen from its weakly allergenic homologue Fra a 3 that was fully digested under all conditions. In contrast, shrimp Pen a 1 allergen was clearly digested by pancreatin into a ladder of truncated and lower molecular mass bands that remained reactive on immunoblot, after pepsin pre-treatment with PPRs 10 and 1, but was completely digested after PPR 0.1. Its pig homologue was highly susceptible to pancreatin digestion irrespective of the preceding pepsin digestion conditions, allowing distinction of the allergenic Pen a 1 from its non-allergenic pig homologue. Ara h 2 showed a quite unexpected behavior during pancreatin digestion after pepsin digestion at pH 1.2, independent of the PPR: Both on SDS-PAGE and on immunoblot, the typical Ara h 2 doublet clearly decreased in the presence of pancreatin at 10 min but increased again to an intensity close to that observed at t = 0. We have no explanation for these observations, but one could speculate that processes like transient aggregation and/or reassembly after clipping of the molecule. Pea PA2 albumin overall displayed a higher resistance to pancreatin than Ara h 2 (Table 2). Overall, one may conclude that, in combination with proposed more physiological conditions of pepsin digestion (pH 2.5–4.0), the addition of a pancreatin phase does not improve the power to discriminate allergens from non-allergens. After an exposure to pepsin at pH 2.5 or 4.0, the stability differences between allergens and non-allergens are inconsistent with no added capability to characterize stability of the protein in the presence of either pepsin or pancreatin.

In summary, this study has challenged the paradigm that there is a straightforward relation between resistance to GI pepsin digestion and allergenicity. Of the four established allergens tested, fish parvalbumin was highly susceptible to pepsin but very resistant to pancreatin. For the other three it was essentially the other way around: quite resistant to pepsin but highly susceptible to pancreatin, in particular if preceded by supposedly more physiological conditions for the pepsin phase. Having said that, neither susceptibility to pepsin nor to pancreatin can characterize whether a candidate protein is likely to be an allergenic hazard. In order to support a revision to the existing low pH, high PPR assay format, the observations that emerged with the pairs of protein would have to be much more consistent than data demonstrate herein. A piece of the puzzle still missing is what role the food matrix may play in determining the digestibility of proteins. It is quite plausible that protein in a matrix may be less accessible to proteolytic enzymes and consequently less susceptible to digestion than a purified protein in aqueous solution.

## Conclusions

The paradigm of resistance to GI proteolysis being a key, but not fully consistent characteristic of allergens does in fact not survive the scrutiny of the present assessment, i.e. not for predicting whether a protein is likely to be or become an allergen. Having said that, resistance to digestion is relevant for the simple reason that relatively higher resistance (both intactness and time to digestion) to pepsin and/or pancreatin will facilitate a higher quantity of any protein, allergen or non-allergen, delivered to the intestinal immune system and potentially cause systemic allergic reactions in those previously sensitized. In this context, the resistance to digestion is in relation to foods consumed. As such, the current practice to evaluate resistance to pepsin digestion is a relevant consideration in the allergenicity risk assessment of novel proteins that should be taken along, mostly as an exposure assessment in the weight-of-evidence approach [28]. The addition of a sequential pancreatin (duodenal) phase to follow the pepsin digestion phase does not improve the power to discriminate allergens from non-allergens. However, with the susceptibility profile of parvalbumin in mind, it is probably valuable to extend the current practice of pepsin digestion protocols (optimal pH and PPR) [29] with a stand-alone pancreatin digestion protocol. Together these separate assays provide a platform to measure a protein’s relative intactness and the rate at which pepsin and pancreatin digest a protein. The assays in this regard support an assessment of whether a protein may survive GI digestion in sufficiently high quantity and integrity to induce systemic reactions.

## Additional files

**Additional file 1: Fig. E1.** Selected SDS-PAGE and immunoblot samples are shown for both albumins, Ara h 2 and pea albumin PA2. Panel a illustrates that pea albumin PA2 is truncated at low PPR but truncated molecules are still detected on immunoblot. Panel b shows gastric digestion of Ara h 2 at low pH and high PPR. Under these conditions the upper band is quite stable but the lower band is truncated. Truncated molecules are not recognized on immunoblot. Panel c and d illustrates that Ara h 2 (panel c) and pea albumin PA2 (panel d) behave differently when gastric digestion at pH 1.2 and pH 2.5 are compared. Ara h 2 is quite stable under both conditions, but if anything slightly more susceptible to digestion at pH 2.5. For pea albumin PA2 this is the other way around. It is clearly more resistant to digestion at pH 2.5 than pH 1.2.

**Additional file 2: Fig. E2.** Selected SDS-PAGE and immunoblot samples are shown for both tropomyosins, from shrimp (Pen a 1) and pig. At pH 1.2 and PPR 0.1 shrimp tropomyosin is fully resistant to pepsin digestion up to 1 h (panel a). At pH 2.5 some truncation is observed, with truncated molecules still being recognized on immunoblot (panel b). For pig tropomyosin, the upper band is not anymore recognized on immunoblot after 60 min of pepsinolysis at pH 1.2 and PPR 0.1 (panel c). Panel d illustrates the susceptibility of shrimp tropomyosin to duodenal digestion after a preceding gastric digestion at pH 1.2/PPR 10: truncated molecules are still detected by rabbit antibodies.

**Additional file 3: Fig. E3.** SDS-PAGE and immunoblot is shown for both bovine collagen after duodenal digestion preceded by gastric digestion at pH 1.2/PPR 0.1. Under these conditions, bovine collagen is fully resistant to duodenal digestion.

## Abbreviations

GI: gastro-intestinal
GM: genetically modified
LTP: lipid transfer protein
PPR: pepsin to protein ratio
SDS-PAGE: sodium dodecyl sulfate polyacrylamide gel electrophoresis
SGF: simulated gastric fluid
