# Supplementary figures and images for: Protease resistance of food proteins: a mixed picture for predicting allergenicity but a useful tool for assessing exposure
Source: Clin Transl Allergy. 2018 Aug 10;8:30. doi: 10.1186/s13601-018-0216-9 (PMC6085708; doi:10.1186/s13601-018-0216-9)

## Slide 1
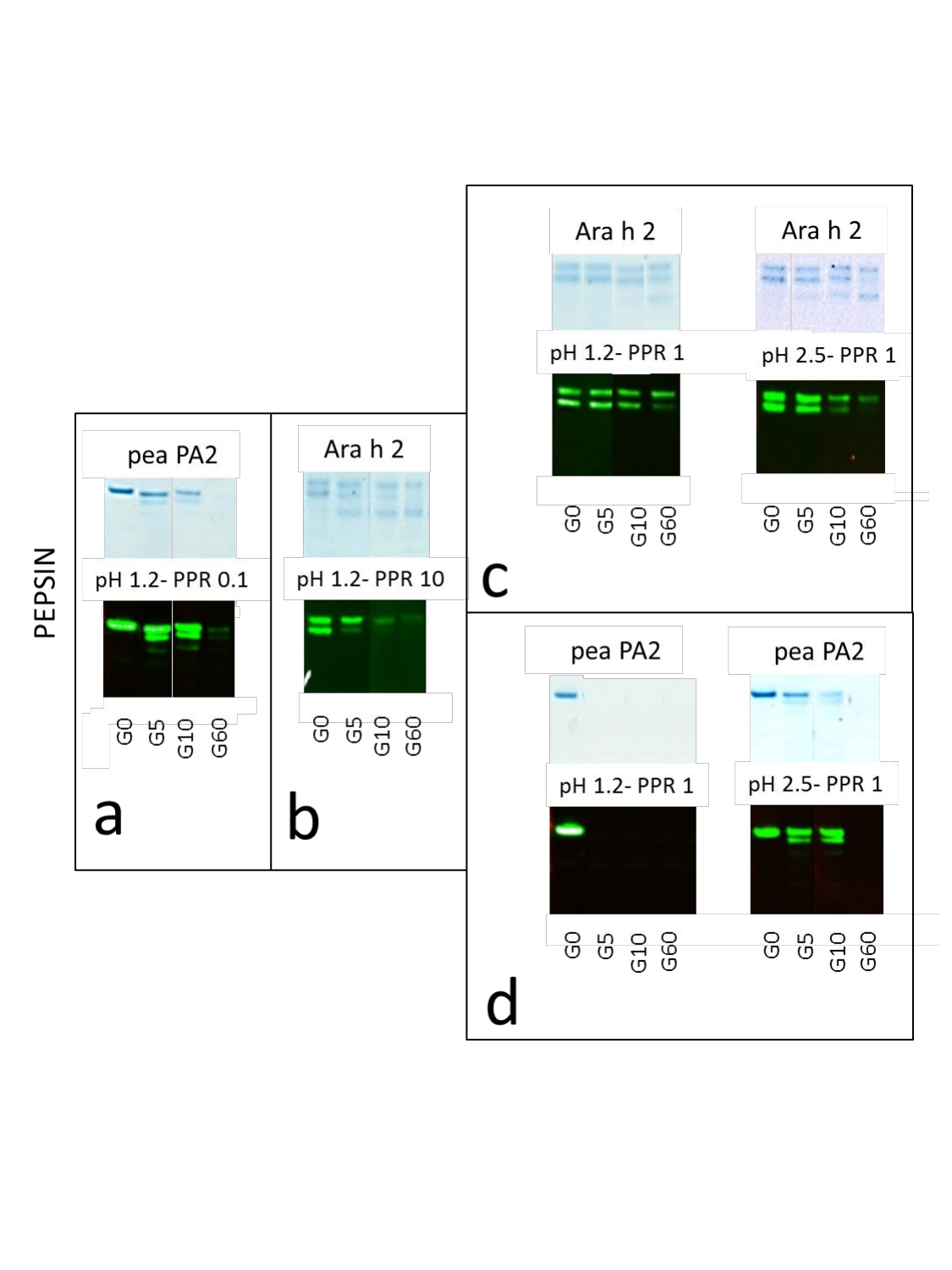

Supplement: Supplementary file 1 — Additional file 1: Fig. E1. Selected SDS-PAGE and immunoblot samples are shown for both albumins, Ara h 2 and pea albumin PA2. Panel a illustrates that pea albumin PA2 is truncated at low PPR but truncated molecules are still detected on immunoblot. Panel b shows gastric digestion of Ara h 2 at low pH and high PPR. Under these conditions the upper band is quite stable but the lower band is truncated. Truncated molecules are not recognized on immunoblot. Panel c and d illustrates that Ara h 2 (panel c) and pea albumin PA2 (panel d) behave differently when gastric digestion at pH 1.2 and pH 2.5 are compared. Ara h 2 is quite stable under both conditions, but if anything slightly more susceptible to digestion at pH 2.5. For pea albumin PA2 this is the other way around. It is clearly more resistant to digestion at pH 2.5 than pH 1.2. [file 13601_2018_216_MOESM1_ESM.pptx]

## Slide 1
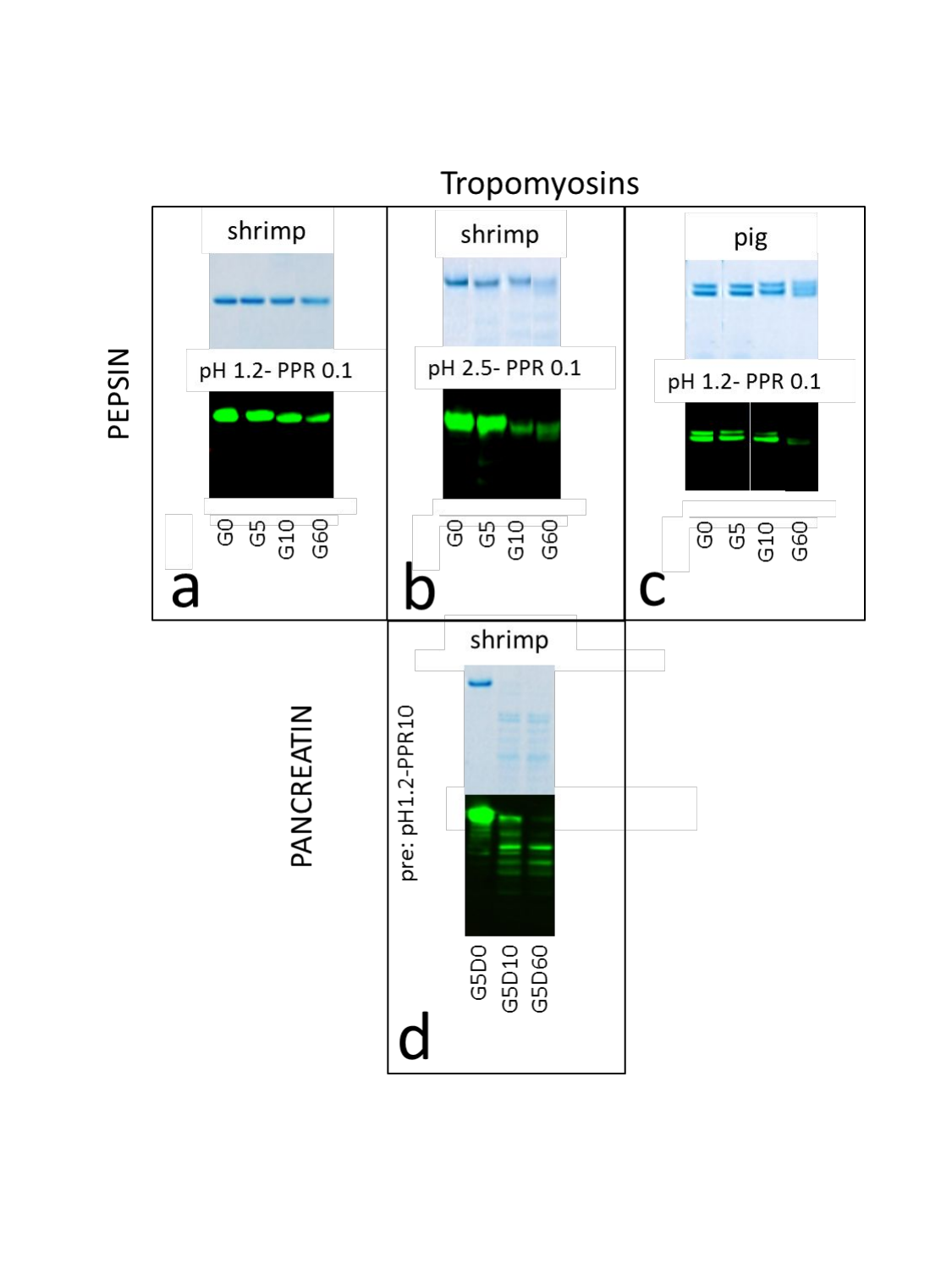

Supplement: Supplementary file 2 — Additional file 2: Fig. E2. Selected SDS-PAGE and immunoblot samples are shown for both tropomyosins, from shrimp (Pen a 1) and pig. At pH 1.2 and PPR 0.1 shrimp tropomyosin is fully resistant to pepsin digestion up to 1 h (panel a). At pH 2.5 some truncation is observed, with truncated molecules still being recognized on immunoblot (panel b). For pig tropomyosin, the upper band is not anymore recognized on immunoblot after 60 min of pepsinolysis at pH 1.2 and PPR 0.1 (panel c). Panel d illustrates the susceptibility of shrimp tropomyosin to duodenal digestion after a preceding gastric digestion at pH 1.2/PPR 10: truncated molecules are still detected by rabbit antibodies. [file 13601_2018_216_MOESM2_ESM.pptx]

## Slide 1
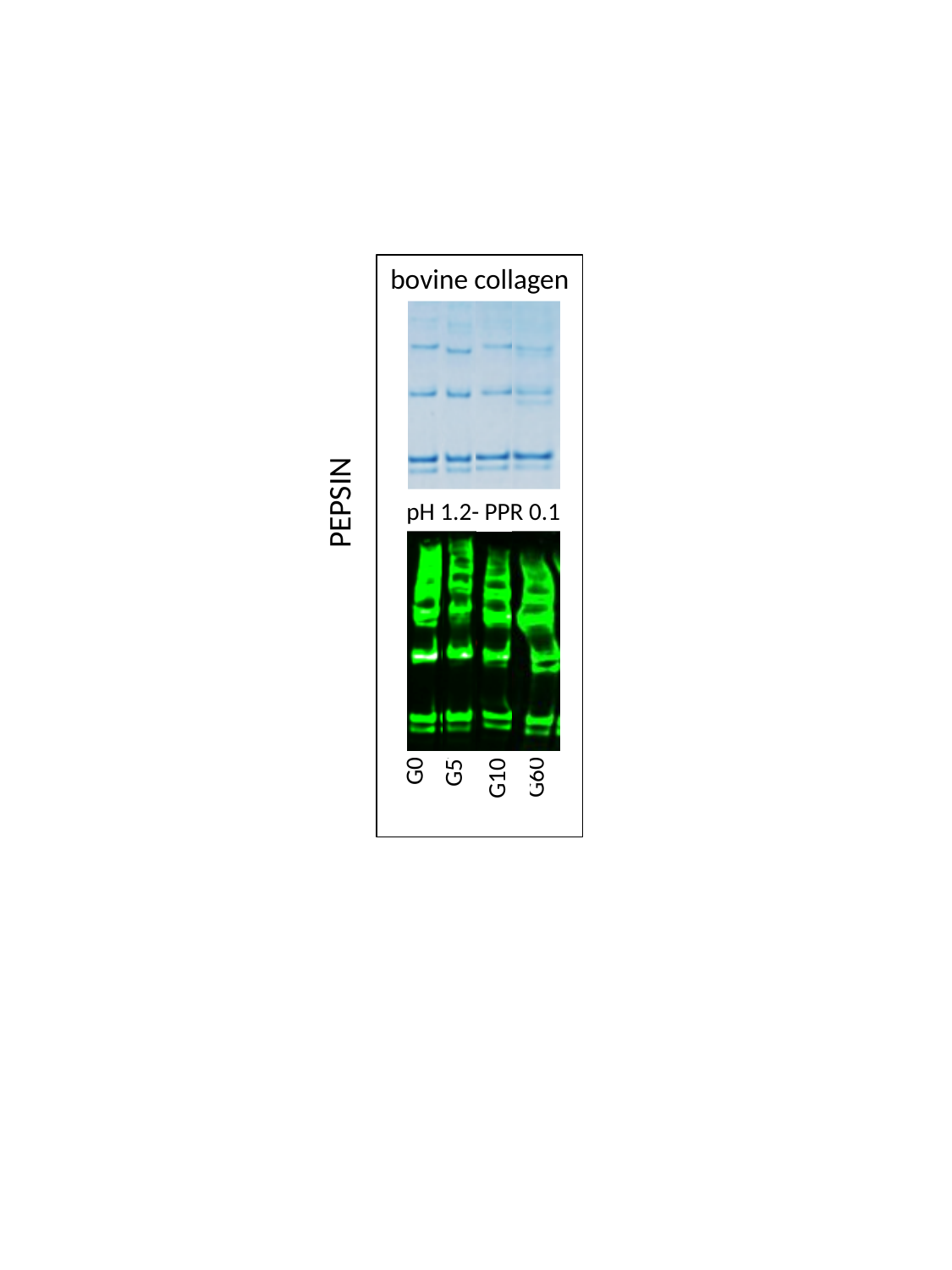

bovine collagen
PEPSIN
pH 1.2- PPR 0.1
G60
G0
G10
G5

Supplement: Supplementary file 3 — Additional file 3: Fig. E3. SDS-PAGE and immunoblot is shown for both bovine collagen after duodenal digestion preceded by gastric digestion at pH 1.2/PPR 0.1. Under these conditions, bovine collagen is fully resistant to duodenal digestion. [file 13601_2018_216_MOESM3_ESM.pptx]
